# Correlation between p53 and Mdm2 expression with histopathological parameters in cattle squamous cell carcinomas

**DOI:** 10.14202/vetworld.2022.10-15

**Published:** 2022-01-07

**Authors:** Waseem Al-Jameel, S. S. Al-Mahmood, A. M. Al-Saidya

**Affiliations:** Department of Pathology and Poultry Diseases, College of Veterinary Medicine, University of Mosul, Mosul, Iraq.

**Keywords:** cattle, immunoexpression, Mdm2, p53, squamous cell carcinoma

## Abstract

**Background and Aim:**

Squamous cell carcinoma (SCC) is the most common form of carcinoma in cattle. Histopathological grading systems have been utilized over several decades for estimating the malignancy of cattle SCCs. This study aimed to detect p53 and Mdm2 expression in different SCC cases in cattle and correlate their expression with the SCC histopathological grading.

**Materials and Methods:**

Cattle SCC cases were collected at the Veterinary Teaching Hospital in Nineveh. The SCC grading system categorized the cases histologically based on their differentiation grade into three groups: Well, moderately, and poorly differentiated. The SCC cases were subsequently verified for p53 and Mdm2 immunoexpression.

**Results:**

Fourteen of 16 examined cattle SCC samples tested positive for p53 expression. Moreover, 15 out of the 16 SCC samples tested positive for Mdm2 expression. The increased immunoreactivity of both p53 and Mdm2 was associated with a poor histological grading of the cattle SCC. There is a positive correlation between the nuclear expression of p53 and Mdm2, and the degree of differentiation and the number of mitotic figures in the examined cattle SCC samples.

**Conclusion:**

Our results demonstrate an increased p53 and Mdm2 expression in cattle SCC cases characterized by poor histopathological grading, thus suggesting an essential role of these molecules in the development of moderately and poorly differentiated SCC in cattle.

## Introduction

Squamous cell carcinoma (SCC) is the leading critical cancer of farm animals. It is commonly seen in cattle, sheep, equines, cats, and, less often, in dogs [1]. In cattle, skin SCC is the most common type of primary cancer of keratinocytes developing from epithelial cells [2]. Moreover, SCC can also be noticed in structures with stratified squamous epithelium and mucocutaneous junctions, such as the eyelids, the vulva, and the vagina [3]. Cattle SCC has been recognized worldwide as a significant cause of income loss due to its diagnosis, which excludes the cattle’s meat from consumption after slaughter [4]. Although many carcinogenic factors are known to cause SCC, prolonged exposure to sunlight and its ultraviolet (UV) radiation seems to be a catalytic factor in developing this type of cancer [5]. Exposure to UV radiation is the leading risk factor for skin SCC in humans [6] and animals [7]. The incidence of SCC is usually linked to prolonged UV light exposure and typically starts from the mucocutaneous junction, especially at the eyelids and the anogenital areas. These areas are poorly pigmented, and the absence of melanocytes defines a photosensitive part in the mucosal and epidermal surfaces [5]. It has been well established that UV radiation can alter proliferation-inducing genes, and these alterations have been experimentally recognized in UV-stimulated SCC cases [8,9].

Like other oncogenes, UV light induces mutation in tumor suppressor genes and oncogenes, and *p53* is the primary specific tumor suppressor gene detected in both human and animal SCC cases [10,11]. The p53 gene plays a key part in the cell cycle, DNA repair, and apoptosis, leading to increased levels of a nuclear phosphoprotein that works as an adverse organizer of cell division. The alteration of *p53* disturbs its normal function or gains abnormal roles for the p53 protein, therefore permitting cancer cells to get away from apoptosis, a critical event in carcinogenesis [12]. Consequently, the expression of the p53 protein increased in the nucleus of cancer cells [9]. Even though of significance, only a few publications focus on the role of p53 in cattle SCC [9,13]. *MDM2*, a normal proto-oncogene, may attach to the *p53* domain to deactivate its transcriptional action. Correspondingly, *p53* may be linked to the *MDM2* intron to stimulate the transcriptional activity of *MDM2*, therefore creating an opposite response that regulates DNA repair and cell proliferation [14]. Both the p53 and the Mdm2 proteins are overexpressed in the last stages of cancer [15]. *MDM2* overexpression is detectable in many cancers with or without a mutation of *p53* [16,17]. Nevertheless, the correlation between the expression of both proteins and cattle SCC histopathological characteristics remains unclear.

Hence, our study aimed to assess whether the expression of Mdm2 and p53 proteins could be of clinicopathological significance for the diagnosis of the severity of cattle SCC.

## Materials and Methods

### Ethical approval

The study was approved by University of Mosul, College of Veterinary Medicine (Approval no. UM.VET.2021.010).

### Study period and location

This study was carried outfrom November 2019 to December 2020 in Mosul, Iraq. The tumors samples were collected from cattle during a surgical procedure at the Veterinary Teaching Hospital in Nineveh Province.

### Samples

Sixteen SCC samples from various cattle breeds were surgically managed by excisional biopsy. Samples were excised from different anatomic locations, for example, vagina, vulva, eye, and eyelids. All samples were fixed in neutral buffered formalin (10%) for up to 48 h at maximum, embedded in wax, and 5 mm sections were stained with hematoxylin and eosin.

### Histopathology

All SCC samples were categorized according to macroscopic and microscopic types. The histological grading system of SCC was based on Broder’s system depending on the grade of keratinization, the island development, and the squamous differentiation of cancer tissues [18]. According to this system, the degree of differentiation was graded as (i) Grade 1 (well-differentiated SCC) that can be distinguished by the presence of multiple big keratin pearls and big islands, with clear squamous differentiation, (ii) Grade 2 (moderately differentiated SCC) that is characterized by the presence of medium-sized pearls and medium islands, with clear squamous differentiation, as well as (iii) Grade 3 (poorly differentiated SCC) that is recognizable by the presence of cancer cells that did not form keratin pearls with separate cell keratinization, rare islands, and poor squamous differentiation. In addition, the grading system of SCC was also evaluated depending on the presented mitotic cell figures [19]. The index of mitosis was assessed as the number of mitotic cell figures for each high-power field (HPF), from 1 to 2 (Grade 1), 3 to 5 (Grade 2), and 6 or above (Grade 3). All slides were reviewed and analyzed independently by three experienced pathologists.

### Immunohistochemistry (IHC)

IHC was achieved using the avidin-biotin immunoperoxidase technique. The adhesive slides were dewaxed and rehydrated. A 3% hydrogen peroxide-methanol solution was used to block the endogenous peroxidase for 30 min. Subsequently, the slides were washed in phosphate-buffered saline (PBS; pH 7), and non-specific proteins were then blocked by blocking solution for 60 min at 25°C (room temperature). The slides were then incubated with primary antibodies (p53 rabbit polyclonal, dilution 1:100, Wuhan Fine Biotech, China; Mdm2 rabbit polyclonal, dilution 1:100, Wuhan Fine Biotech) overnight at 40°C. After washing with PBS, the slides were incubated with poly-horseradish peroxidase goat anti-rabbit immunoglobulin G (dilution 1:100, Wuhan Fine Biotech) for 1 h at 37°C. After another PBS washing, the reaction was amplified using an avidin-biotin complex. The slides were counterstained with hematoxylin, rinsed in distilled water, dehydrated, and coverslipped. Slides of breast cancer, known to show strong p53 and Mdm2 expression [20], were used as positive immunohistochemical controls for both antibodies. For the negative control, non-immune serum was used instead of the primary antibodies. The other steps were the same.

Nuclear labeling was assessed as a positive indicator for p53 and Mdm2 expression. The expression of p53 and Mdm2 was assessed under a microscope at 200×. Using the mean labeling index as explained before for p53 [21] and Mdm2 [16], the expression of both proteins was established by detecting 500 nuclei of cancer cells in the area with the highest labeling density. For both p53 and Mdm2, the expression score was measured using a 4-point grading system as follows: 0 (<10%), 1 (10-20%), 2 (20-50%), and 3 (>50%). Cancer samples were considered positive if their estimated score was either 2 or 3. All slides were reviewed and analyzed independently by three experienced pathologists.

## Results

**Figure-1:**
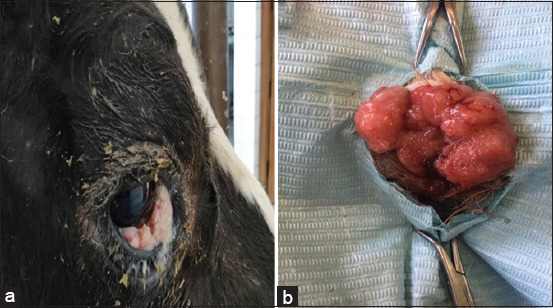
Squamous cell carcinoma (SCC) in cattle. (a) SCC in the eyelid appears as a cauliflower-like appearance. (b) SCC in vulvar mucosa appears as papillary projections.

All SCC samples were surgically collected from different cattle breeds aged between 5 and 9 years old. Six cancer samples had been excised from eyelids, five from the vagina, three from the eye, and two from the vulva (Table-1 and Figure-1). All types of SCC, either arising from skin keratinocytes or mucosal keratinocytes, had the same histopathological characters. The current study was conducted on 16 samples of different SCCs that were divided into three groups based on their level of differentiation: Well differentiated, moderately differentiated, and poorly differentiated (Table-1). Their grading depended on the degree of similarity of the cancer cells to the normal epithelium and on their ability to form keratin pearls. This study revealed that 6 samples (37.5%) were of Grade 1 SCC, 4 samples (25%) were of Grade 2 SCC, and 6 samples (37.5%) were of Grade 3 SCC.

**Table 1:** Association of immunohistochemical expression of p53 and Mdm2 with histopathological characteristics in cattle SCC samples.

Microscopic characters		p53 expression***	Mdm2 expression***	Macroscopic characters
			
Differentiation*	Mitotic figures**	Score	Score	Location
+++	3	2	3	Eyelids
+++	2	3	3	Eyelids
+	1	1	1	Eyelids
++	2	2	2	Eyelids
+	1	0	1	Eyelids
++	2	1	2	Eyelids
++	2	3	2	Vagina
+++	2	2	3	Vagina
+	1	1	1	Vagina
+++	3	3	2	Vagina
+	1	1	1	Vagina
+++	2	3	2	Eye
++	2	2	3	Eye
+	1	1	1	Eye
+	1	0	0	Vulva
+++	3	2	2	Vulva

* (+++) Poorly differentiated SCC; (++) moderately differentiated SCC; (+) well-differentiated SCC.

** Mitotic cell figures from 0 to 2=(1); 3 to 5=(2); >6=(3).

*** Expression score: 0 (<10%); 1 (5-20%); 2 (20-50); 3 (>50%). SCC=Squamous cell carcinoma

**Figure-2:**
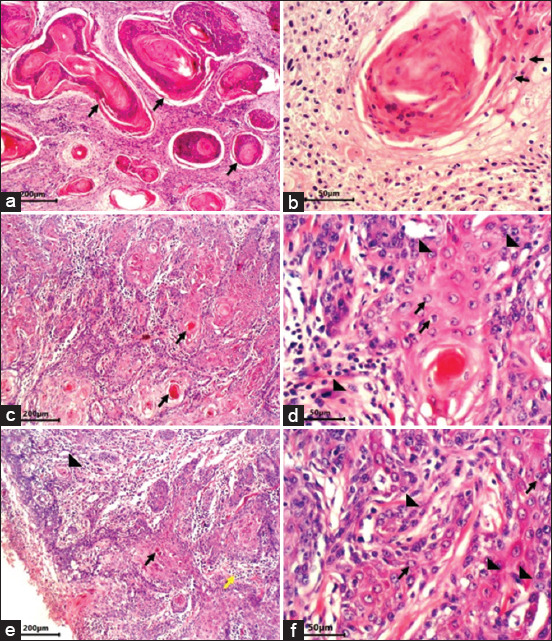
Histological characteristic of squamous cell carcinoma (SCC), cattle, and skin. (a) Well-differentiated SCC with widespread of different shapes and sizes islands with keratinous pearls in the center (arrows) (hematoxylin and eosin [H&E], 200 µm). (b) Well-differentiated SCC, histological aspect of a keratin pearl with hyperplasia of keratinocytes (arrows) and rare mitotic figures (H&E, 50 µm). (c) Moderately differentiated SCC, a significantly less keratin formation with small-sized keratin pearls (arrows) (H&E, 200 µm). (d) Moderately differentiated SCC showing vesicular nuclei (arrows) with mitotic rate of five cells in high-power field (HPF) (arrowheads) (H&E, 50 µm). (e) Poorly differentiated SCC showing separate cell keratinization (arrow), rare small islands (arrowhead), and poorly differentiated cells (yellow arrow) (H&E, 200 µm). (f) Poorly differentiated SCC showing cells with enlarged nuclei, one-to-two prominent nucleoli, well-defined cell borders (arrows) and with mitotic rate of 6-10 cells in HPF (arrowhead) (H&E, 50 µm).

Well-differentiated SCC samples consist of fully differentiated squamous cells, organized in islands of different shapes and sizes, bearing keratinous pearls in the center. Inside these keratinous pearls, the cells are acidophilic with different shapes and sizes of nuclei. Some of them might bear different pyknotic and karyolitic nuclei (Figures-2a and b). The intermediate grade refers to moderately differentiated SCC with significantly less keratin formation, demonstrating small-sized keratin pearls with an augmented number of poorly differentiated cells. The nuclei of the cancer cells of this group of SCC have different sizes and shapes and appear with large nucleoli (Figures-2c and d). The poor grade refers to cancers with simple, separate cell keratinization, rare tiny islands, and characterized by poorly differentiated cells. The cancer cells of this third group of SCCs are heterogeneous and interact with each other and the extracellular matrix (Figures-2e and f).

The mitotic index was the lowest in the well-differentiated SCC samples (0-2 cells in each HPF) (Figure-2b). In the moderately differentiated SCC samples, the mitotic index was around five cells in each HPF (Figure-2d), while in poorly differentiated SCC samples, the mitotic index was the highest (6-10 cells in each HPF) (Figure-2f).

**Figure-3:**
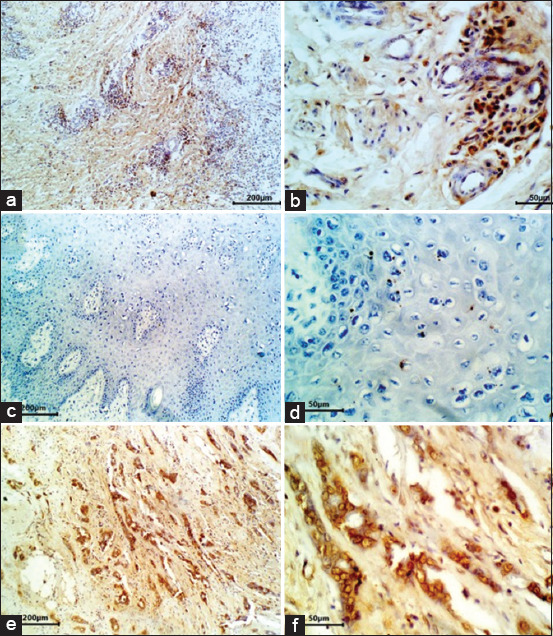
Immunohistochemical stains for P53. (a) Poorly differentiated SCC. Nearly all cancer cells nuclei have high immunoreactivity for p53 protein (IHC, 200 µm). (b) P53-positive cancer cells were located generally in around the island in poorly differentiated SCC (IHC, 50 µm). (c) Poorly differentiated SCC. Negative control removed p53 antibody but involved all other steps (IHC, 200 µm). (d) Poorly differentiated SCC. Negative control removed p53 antibody but involved all other steps (IHC, 50 µm). (e) Human breast cancer, which highly expresses nuclear p53 protein, is used as the positive control (IHC, 200 µm). (f) Human breast cancer, which highly expresses p53 protein, is used as the positive control (IHC, 50 µm).

Depending on the obtained immunohistochemical results, a 4-point grading system was applied based on the staining strength and the proportion of positive cells (Table-1). The expression of both 53 and Mdm2 was localized mainly in the nucleus of the cancer cells. Among the 16 SCC samples, there was a substantial difference in the positive expression of p53 and Mdm2 between the moderately and the poorly differentiated samples compared to the negative expression of the well-differentiated samples. Regarding the expression of p53, 9 samples (56.3%) were identified as immunoreactive, all of which were either moderately or poorly differentiated. From these nine positive samples, five were scored as “2” and four were scored as “3” in the 4-point expression scale. Of the other seven samples, five were scored as “1” and two were scored as “0” in the 4-point expression scale (Table-1). The p53-positive cancer cells were located mainly around the cancer island, especially in the poorly differentiated samples, with a diffuse staining pattern (Figures-3a and b). By replacing the p53 antibody with a non-immune serum, all samples that were stained with a negative control have failed to show immunoreactivity (Figures-3c and d).

**Figure-4:**
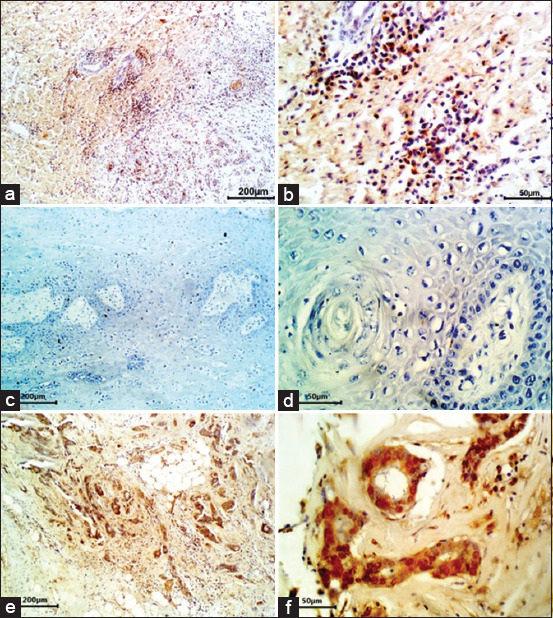
Immunohistochemical stains for Mdm2. (a) Poorly differentiated squamous cell carcinoma (SCC). Nearly all of cancer cells nuclei have high immunoreactivity for Mdm2. Immunohistochemistry (IHC) (200 µm). (b) Mdm2-positive cancer cells were located generally around the island in poorly differentiated SCC (IHC, 50 µm). (c) Poorly differentiated SCC. Negative control removed the Mdm2 antibody but involved all other steps (IHC, 200 µm). (d) Poorly differentiated SCC. Negative control removed Mdm2 antibody but involved all other steps (IHC, 50 µm). (e) Human breast cancer, which highly expresses nuclear Mdm2, is used as the positive control (IHC, 200 µm). (f) Human breast cancer, which highly expresses Mdm2, is used as the positive control (IHC, 50 µm).

On the other hand, the human breast cancer samples demonstrated intense staining as positive controls (Figures-3e and f). Regarding the Mdm2 staining, 10 samples (62.5%) were immunoreactive, and all of them were either moderately or poorly differentiated. Higher numbers of labeled cells were observed in these 10 Mdm2-positive samples in the majority of the moderately differentiated samples. The other six samples corresponded to well-differentiated samples (Table-1). The Mdm2-positive cancer cells were located mainly around the cancer island, especially in the poorly differentiated samples (Figures-4a and b). All samples stained with the negative control by replacing the Mdm2 antibody with a non-immune serum have failed to show any immunoreactivity (Figures-4c and d). On the other hand, the human breast cancer samples demonstrated intense staining as positive controls (Figures-4e and f).

## Discussion

SCC is a common malignant tumor of cattle originating from the squamous epithelium in various locations. The most common locations for the cattle SCC are the eyelids, the vagina, the eyes, and the vulva. The breeding system of cattle that involves a whole-year-round exposure of the animals to UV radiation allows for prolonged exposure to the sunlight’s oncogenic activity, as described by others in different parts of the world [5,22]. Hence, the SCC cases are primarily detected in adult cattle [22]. Excessive exposure to UV radiation can induce DNA damage and cause mutations in vulnerable genes, leading to SCC development [23]. In human SCC, *p53* and *MDM2* mutations are identified in most cases [24]. The current study has investigated the immunohistochemical expression of p53 and Mdm2 in cattle SCC cases. Our results have indicated that both p53 and Mdm2 are highly expressed in the poorly and moderately differentiated cattle SCC samples compared to the absence of their expression in well-differentiated samples. Moreover, our study has identified that higher percentages of p53 and Mdm2 expression levels are associated with a more poorly differentiated SCC.

In the current study, immunohistochemical detection has displayed an increased expression of the p53 protein in the cancer cells’ nuclei of the SCC samples. In addition, Mdm2 antibodies have revealed an increased expression of the protein in the nuclei of SCC cancer cells. These findings may be of significant pathogenic importance, as high Mdm2 nuclear expression could inhibit the p53 function and, thus, endorse unlimited cell cycling. The interaction between Mdm2 and p53 could suppress the p53’s transcriptional role and lead to the removal of excess p53 through proteolysis [14,25]. As p53 is considered a tumor suppressor gene, the Mdm2 overexpression could stimulate cell proliferation by inhibiting the p53-mediated elimination of the DNA damage and cell cycle [25,26].

The current study measured the p53 and the Mdm2 immunohistochemical expressions in cattle SCC samples and correlated these expressions with the samples’ histopathological characteristics. We have, herein, established that the p53 protein expression was significantly higher around the cancer island of the cattle SCC, especially in the poorly differentiated samples with diffuse staining patterns. Our findings are in agreement with those of others [9,19]. The expression of p53 was considerably greater in the poorly differentiated SCC samples than in those of well or moderately differentiated SCC. Based on what we know so far, these findings are novel. On the contrary, until now, it was only recognized that the growth of the poorly differentiated cattle SCC was simply dependent on time and the strength of the preceding UV light exposure [27].

In addition, in this study, the Mdm2 expression was significantly higher around the cancer island of the cattle SCC, especially in the poorly differentiated samples. Interestingly, there has been no report on the expression of Mdm2 in cattle SCC. However, the enhanced expression of Mdm2 is the outcome of the overexpression of p53 in different types of cancer [28]. Mdm2 can interact with p53 and can deactivate it [14]. Therefore, the p53 and Mdm2 expression levels are raised in cancer cells. In this case, Mdm2 has a cooperative role in p53 as it can interrupt the cell cycle [29]. As Mdm2 follows p53, the increased expression of p53 in the cattle SCC might justify the increased expression of Mdm2 [27].

Interestingly, in cattle SCC, a relationship exists between the p53 and the Mdm2 expression, as described in human SCC [16]. In 14 out of the 16 examined samples (87.5%) of cattle SCC, the p53 expression was identified as positive, a rate that is moderately higher than that of an earlier study (67% positive) [19]. Moreover, 15 out of the 16 examined samples (93.7%) of cattle SCC were identified for the 1^st^ time in the literature as positive for Mdm2. Furthermore, our results suggested that the expression of both p53 and Mdm2 increases and is associated with a poor histological grading of the cattle SCC. These results demonstrate that the p53 and the Mdm2 overexpression in cattle SCC might be involved in the SCC carcinogenesis. There could be a connection between the grade of SCC differentiation and the degree of increased expression of p53 and Mdm2 in cattle SCC.

## Conclusion

Our results indicate an elevated amount of p53 and Mdm2 immunoreactivity in cattle SCC cases, which suggest an essential role of these molecules, particularly in the carcinogenesis of the moderately and poorly differentiated cases. Moreover, the immunohistochemical expressions of p53 and Mdm2 could be important markers of carcinogenesis for cattle SCC, which could indicate the aggressive state of these lesions.

## Authors’ Contributions

WA, SSA, and AAA: Performed the study (samples collection, tissue processing, designed the experiment, and analyzed the data). SAA: Carried out the histology. WA: Carried out the IHC. All authors read and approved the final manuscript.
